# Analysis of the Influencing Factors of Tumor Volume, Body Immunity, and Poor Prognosis after ^125^I Particle Therapy for Differentiated Thyroid Cancer

**DOI:** 10.1155/2023/8130422

**Published:** 2023-05-03

**Authors:** Liling Tan, Zhijun Chen, Wenjun Wang, Yu Su, Zhen Wu, Ling Yi, Zhipeng Zheng

**Affiliations:** ^1^Department of Nuclear Medicine, The Second Affiliated Hospital, Nanchang University, Nanchang, Jiangxi, China; ^2^Department of Nuclear Medicine, Jiangxi Tumor Hospital, Nanchang, Jiangxi, China

## Abstract

**Objective:**

To analyze the influencing factors of tumor volume, body immunity, and poor prognosis after ^125^I particle therapy for differentiated thyroid cancer.

**Methods:**

A total of 104 patients with differentiated TC who were treated with ^125^I particles during January 2020 to January 2021 was picked. These subjects were graded as low-dose group (80Gy-110Gy) and high-dose group (110Gy-140Gy) according to the minimum dose received by 90% of the target volume (D90) after surgery. The tumor volume before and after treatment was compared, and fasting venous blood was collected before and after treatment. The content of thyroglobulin (Tg) was detected by electrochemiluminescence immunoassay. The levels of absolute lymphocyte count (ALC), lymphocytes, neutrophils, and monocytes were detected on automatic blood cell analyzer. The lymphocyte to monocyte ratio (LMR), neutrophil to lymphocyte ratio (NLR) and platelet to lymphocyte ration (PLR) were calculated. The changes in the condition of patients were closely observed, and the occurrence of adverse reactions in the two groups were compared. The risk factors influencing the efficacy of ^125^I particle therapy for differentiated TC were analyzed through multivariate logistic regression analysis.

**Results:**

The total effective rate of patients in the low- and high-dose groups was 78.85% and 82.69%, respectively (*P* > 0.05). Compared with the pretreatment period, the tumor volume and Tg level in both groups were much lower (*P* < 0.05), and the differences in tumor volume and Tg level had no statistically significant difference between the two groups before and after treatment (*P* > 0.05). At 1 week of the treatment, the total incidence of adverse reactions such as nausea, radiation gastritis, radiation parotitis, and neck discomfort was obviously higher in the high-dose group than in the low-dose group (*P* < 0.05). At 1 month of treatment, the incidence of adverse reactions such as nausea was markedly higher in the high-dose group than in the low-dose group (*P* < 0.05). After treatment, serum NLR and PLR contents were memorably elevated and LMR level was sharply decreased in both groups, and serum NLR and PLR contents were higher and LMR content was lower in the high-dose group than in the low-dose group (*P* < 0.05). Multivariate logistic regression analysis showed that the pathological type of follicular adenocarcinoma, tumor size ≥ 2 cm, clinical stage of III~IV, distant metastasis, and high TSH level before ^125^I particle treatment were all risk factors related to the efficacy of ^125^I particle treatment of TC (*P* < 0.05).

**Conclusion:**

The efficacy of low-dose and high-dose ^125^I particles in the treatment of differentiated thyroid cancer is comparable, among which low-dose ^125^I particles have fewer adverse effects and have less impact on the immunity of the body, which is well tolerated by patients and can be widely used in clinical practice. In addition, the pathological type of follicular adenocarcinoma, tumor size ≥ 2 cm, clinical stage III~IV, distant metastasis, and high TSH level before ^125^I particle treatment are all risk factors that affect the poor effect of ^125^I particles on thyroid cancer treatment, and early monitoring of the above index changes can help evaluate the prognosis.

## 1. Introduction

Thyroid cancer (TC) is one of the most common malignant tumors in the endocrine system, accounting for more than 90% of all endocrine malignant tumors. In recent years, the incidence rate of TC is increasing year by year, which has a certain relationship with region, race, and gender [1]. Previous studies suggested that the incidence of TC was closely related to excessive iodine diet, radiation, abnormal secretion of sex hormones, and thyroid stimulating hormones [2]. With the acceleration of life rhythm and the increase of life pressure in recent years, the number of patients with TC has increased year by year, and most of them are differentiated TC, which accounts for more than 90% of TC. Differentiated TC includes papillary TC and follicular TC, of which papillary TC accounts for about 75%, with slow growth, low malignancy, and high 10-year survival rate. Follicular TC is highly malignant and can metastasize to bone and lung through blood with poor prognosis and the 10-year survival rate of less than 40%. Therefore, effective therapy for differentiated TC is the focus of thyroid related research [3, 4].

^125^I particle is a radioactive particle with wide clinical application at present, which can continuously emit *γ*-ray for a long time to suppress the reproduce ability of tumor cells, thus inhibiting tumor progression. At present, ^125^I particle has a good effect in the treatment of prostate cancer, lung cancer, pancreatic cancer, and other solid tumors [5, 6]. Some scholars found that ^125^I particle therapy could alleviate the clinical symptoms related to lymph node metastasis in patients with refractory differentiated TC with effective and safe local control effect in tumor for short term [7]. However, there are few studies on the effects of different ^125^I particle treatment dose parameters on the patients.

In this study, 104 patients with differentiated TC admitted in our hospital during January 2020 to January 2021 were picked as the subjects to analyze the factors influencing tumor volume, body immunity, and poor prognosis after ^125^I particle therapy for differentiated TC.

## 2. Materials and Methods

### 2.1. General Materials

A total of 104 patients with differentiated TC admitted in our hospital during January 2020 to January 2021 was picked. Inclusive criteria are as follows: (1) all patients were diagnosed as differentiated TC by preoperative pathological examination [8] and underwent total thyroidectomy or subtotal thyroidectomy. (2) All patients received ^125^I particle therapy after operation. (3) The patients and their family members signed the informed consent form and could cooperate with the examination and treatment with good compliance. Exclusion criteria are as follows: (1) the patients with serious functional disorder of important organs. (2) The patients with ^125^I contraindication. (3) The patients complicated with endocrine metabolism and immune system diseases. (4) The patients with predicted survival time of less than 6 months. (5) The patients combined with other malignant tumors. (6) The patient in lactating or pregnant period. These subjects were graded as low-dose group (80Gy-110Gy) and high-dose group (110Gy-140Gy) according to the minimum dose received by 90% of the target volume (D90) after surgery. 52 patients (23 males and 29 females) were graded as the low-dose group, with an average age of (44.85 ± 5.96) years, an average BMI of (21.12 ± 1.45) kg/m^2^, and an average course of disease of (4.52 ± 1.23) years. 52 patients (21 males and 31 females) were graded as the high-dose group, with an average age of (45.15 ± 6.10) years, an average BMI of (20.85 ± 1.33) kg/m^2^, and an average course of disease of (4.33 ± 1.28) years. There existed no significant difference in age, gender, BMI, and other general data between the groups (*P* > 0.05). This experiment was approved by the Ethics Committee of our hospital.

### 2.2. Methods

All patients were treated with ^125^I particle therapy after total or subtotal thyroidectom. (1) Preoperative preparation: blood routine test, biochemical test, thyroid function test, CT, ECG, and ^131^I whole body imaging were performed before operation. Fasting and water deprivation for 4 hours before operation and venous channels were established. (2) Three-dimensional treatment planning system (TPS) plan: this plan is currently an important tool for radiation therapy for oncology, and the planning system can meet the requirements of conventional radiation therapy. The radioactive particle implantation treatment plan through image pictures was designed, and the gross tumor volume (GTV) and planning target volume (PTV) were outlined. The ^125^I particles with an activity of 14.8-25.9 MBq was selected, and the prescription dose was set as 100-150Gy. The quantity, distribution, and puncture needle layout of ^125^I particles were reasonably designed. ^125^I particle was implanted under the guidance of CT, and the CT images were observed after operation for verification. (3) Operation: the patient was guided to take a comfortable position. The puncture point was marked and routine disinfection was conducted. Sterile sheet was paved and local anesthesia was performed using 2% lidocaine. The position of puncture needle was confirmed through CT after puncture. Implantation was conducted with particle spacing of 0.5-1.0 cm and row spacing of 1.0 cm. After CT reexamination and confirmation of correct position, pressure bandage was performed, and antibiotics were routinely used after operation.

### 2.3. Outcome Measures

#### 2.3.1. Efficacy

The efficacy was graded as complete response (CR), partial response (PR), no change (NC), and progression (PD). The lesions disappeared completely, and only strip shadow or no abnormality in imaging were CR. PR referred to the reduction of lesion volume ≥ 50% compared with that before treatment. NC referred to that the lesion volume reduced by <50% or increased by <25% compared with that before treatment. The volume of lesion increased by ≥25% than that before treatment or new lesion appeared was PD. Total effective = CR + PR + NC.

#### 2.3.2. Tumor Volume

Cervical ultrasound was performed before and 3 months after treatment to measure the longitude of thyroid tissue before and after treatment, and the thyroid volume was calculated by Brunn's formula.

#### 2.3.3. Serum Index

The fasting venous blood in the morning before treatment and 3 months after the treatment was collected and centrifuged at 3000 r/min for 10 minutes. The serum was carefully collected and stored at -40°C to avoid repeated freezing and thawing. The content of thyroglobulin (Tg) was detected by electrochemiluminescence immunoassay. The levels of absolute lymphocyte count (ALC), lymphocytes, neutrophils, and monocytes were detected on automatic blood cell analyzer. The lymphocyte to monocyte ratio (LMR), neutrophil to lymphocyte ratio (NLR), and platelet to lymphocyte ration (PLR) were calculated.

#### 2.3.4. Adverse Reactions

The changes in the condition of patients were closely observed, and the occurrence of adverse reactions such as nausea, radiation gastritis, radiation mumps, and neck discomfort in two groups were compared.

#### 2.3.5. Collection of Clinical Characteristics

The indicators such as the patients' age (<45 years old, ≥45 years old), gender (male, female), pathological type (papillary adenocarcinoma, follicular adenocarcinoma), tumor size (<2 cm, ≥2 cm), number of lesions (single, multiple), invasion (no invasion of capsule, membrane invasion, slight invasion outside the thyroid, and obvious invasion outside the thyroid), clinical stage (I~II, III~IV), distant metastasis (yes, no), and the level of thyroid stimulating hormone (TSH) before ^125^I particle therapy (<30 mIU/L, 30-59 mIU/L, 60-89 mIU/L, and ≥90 mIU/L) were collected.

### 2.4. Statistical Analysis

The experimental data were analyzed by SPSS20.0 software. NLR, PLR, LMR, and other measurement data were expressed in $\left(\bar{x}\pm s\right)$ and were compared using t-test between groups; The enumeration data of curative effect and adverse reaction were expressed in (%) and were compared by *χ*^2^ text. The risk factors related to the efficacy of ^125^I particle therapy for differentiated TC were analyzed by multivariate logistic regression analysis. *P* < 0.05 indicated that the statistical results were statistically significant.

## 3. Results

### 3.1. General Data of 104 Patients

**Figure 1:**
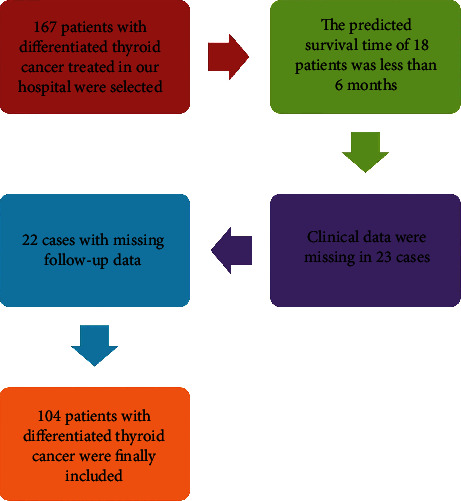
The inclusion process of the general data of 104 patients.

There were 167 cases of differentiated TC included, and 104 patients were finally included after screening according to the inclusion and exclusion criteria. The specific process was shown in Figure 1.

### 3.2. Comparison of Curative Effect

**Figure 2:**
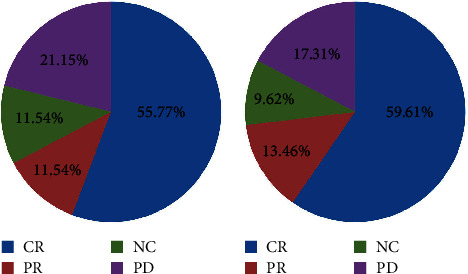
Pie chart analysis of comparison of curative effect between two groups. (a) Low-dose group. (b) High-dose group. Note: CR: complete response; PR: partial response; NC: no change; PD: progression.

The proportions of CR patients in low- and high-dose groups were 55.77% and 59.62%, PR patients of 11.54% and 13.46%, and NC patients of 11.54%, 9.62%, respectively. The total effective rate of patients in the low- and high-dose groups was 78.85% and 82.69%, respectively (*P* > 0.05; Table 1 and Figure 2).

### 3.3. Changes of Tumor Volume

**Figure 3:**
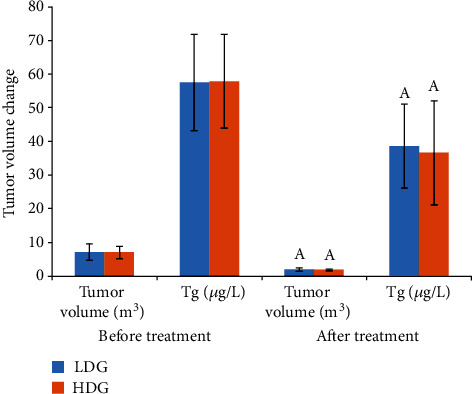
The tumor volume changed after different radiation doses in the two groups. Note: ^A^*P* < 0.05 compared with the same group before treatment.

Compared with the pretreatment period, the tumor volume and Tg level in both groups were much lower (*P* < 0.05), and the differences in tumor volume and Tg level had no statistically significant difference between the two groups before and after treatment (*P* > 0.05; Table 2 and Figure 3).

### 3.4. Comparison of Adverse Reactions

**Figure 4:**
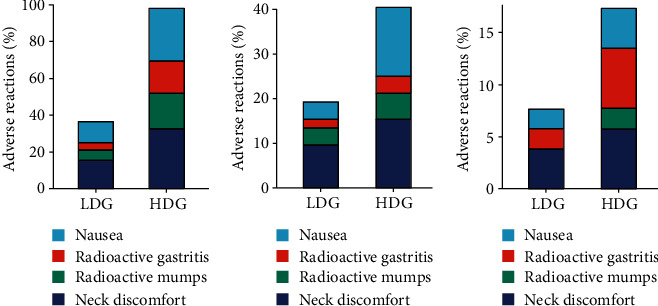
Comparison of the incidence of adverse reactions after different radiotherapy in the two groups. (a) 1 week after treatment. (b) 1 month after treatment. (c) 3 months after treatment.

At 1 week of treatment, the total incidence of adverse reactions such as nausea, radiation gastritis, radiation parotitis, and neck discomfort was obviously higher in the high-dose group than in the low-dose group (*P* < 0.05). At 1 month of treatment, the incidence of adverse reactions such as nausea was markedly higher in the high-dose group than in the low-dose group (*P* < 0.05) but there existed no significant difference in the probability of adverse reactions such as radiation gastritis, radiation mumps, and neck discomfort (*P* > 0.05). At 3 months of treatment, there existed no significant difference in the probability of adverse reactions such as nausea, radiation gastritis, radiation mumps, and neck discomfort between two groups (*P* > 0.05; Table 3 and Figure 4).

### 3.5. Comparison of Immune Function

There existed no significant difference in serum ALC, NLR, LMR, and PLR levels between the two groups before treatment (*P* > 0.05). After treatment, serum NLR and PLR content was memorably elevated and LMR level was sharply decreased in both groups, and serum NLR and PLR contents were higher and LMR content was lower in the high-dose group than in the low-dose group (*P* < 0.05; Table 4).

### 3.6. Univariate Analysis of Factors Influencing the Efficacy of ^125^I Particle Therapy for Differentiated TC

As shown in Table 5, the univariate analysis showed that there existed statistically significant differences between the effective group and the ineffective group in terms of pathological type, tumor size, clinical stage, distant metastasis, and TSH level before ^125^I particle therapy (*P* < 0.05; Table 5).

### 3.7. Risk Factors Related to the Efficacy of ^125^I Particle Treatment Analyzed by Multivariate Logistic Regression Analysis

The indicators with statistical significance in Table 5 were included into the multivariate logistic regression analysis. The results showed that the pathological type of follicular adenocarcinoma, tumor size ≥2 cm, clinical stage of III~IV, distant metastasis, and high TSH level before ^125^I particle treatment were all risk factors related to the efficacy of ^125^I particle treatment of TC (*P* < 0.05; Table 6).

## 4. Discussion

Differentiated TC is the TC with the highest incidence rate at present, which has the characteristics of high differentiation, low malignancy, and better surgical treatment. However, different clinical manifestations of patients, including tumor size, extraglandular invasion, cervical lymph node metastasis, and distant metastasis, will lead to different prognosis of patients. In addition, due to the complex anatomical structure of the thyroid region in the neck, the incidence of early thyroid infiltration or invasion of surrounding tissues is high, and there may be residual thyroid cancer tissue after surgery [9]. Therefore, differentiated TC has a high rate of recurrence, local or distant metastasis. Statistics show that 10%-30% of patients are accompanied by recurrence and metastasis after surgery. If the lung metastasis of thyroid cancer could be diagnosed early and treated effectively, the 10-year survival rate of lung metastasis could be as high as 90% [10]. Therefore, how to reduce the recurrence and metastasis rate of differentiated TC after surgery has become the research focus of medical scholars.

^125^I particle therapy is a new radiation therapy technology in recent years, which effectively protects parathyroid gland, recurrent laryngeal nerve, and other important organs by implanting ^125^I particle into the focus by minimally invasive method without surgical incision and suture. At the same time, ^125^I particles have the characteristics of high local dose and high dose of surrounding normal tissues in brachytherapy, which can effectively reduce the impact on normal thyroid tissues while ensuring the efficacy [11, 12]. At present, ^125^I particles have achieved good efficacy in the treatment of TC, non-small-cell lung cancer and other malignant tumors [13, 14], but there is no formal research report on dosage application. There are few studies on the effects of different doses on the efficacy, adverse reactions, and immunity of differentiated TC. It was found that compared with before treatment, the lymph node metastasis was much smaller; the Tg level and postoperative dose parameters were much lower after treatment when ^125^I radioactive particles were implanted for refractory differentiated TC treatment [15], suggesting that ^125^I radioactive particle implantation could achieve the expected dose distribution and effectively control tumor progression. In this study, the total effective rate of patients in the low- and high-dose groups was 78.85% and 82.69%, respectively. Compared with the pretreatment period, the tumor volume and Tg level in both groups were much lower. The results of this study suggested that ^125^I particles with D90 in the range of 80Gy-140Gy were effective in the treatment of differentiated TC, which could effectively inhibit tumor progression. The effects of low dose and high dose after surgery were similar.

Radiotherapy treats tumors using radiation, usually accompanied by side effects such as nausea, vomiting, and gastrointestinal dysfunction, which not only increases the pain of patients but also has a certain impact on their lives. Long term adverse reactions further reduce the patient's tolerance [16, 17]. Therefore, how to reduce the side effects of radiotherapy and increase the confidence of patients to overcome the disease are also important options for selecting treatment plans. Radiotherapy can not only inhibit the proliferation of tumor cells but also inhibit the immune function of the body. NLR and PLR are commonly used clinical immune indicators, and the increased content of NLR and PLR usually indicates that the body is in an immunosuppressive state [18]. LMR is a marker of inflammatory immune response, and decreased level of LMR indicates the malignant progress of tumor. In this experiment, the high-dose group had much higher probability of nausea, radiation gastritis, radiation mumps, and neck discomfort than the low-dose group at 1 week after treatment. At the first month after treatment, the probability of nausea in the high-dose group was markedly higher than that in the low-dose group. After treatment, serum NLR and PLR content was higher and LMR content was lower in the high-dose group than in the low-dose group. Hammad et al. [19] showed that radiotherapy could reduce lymphocyte count, and lymph nodes were the key to regulating tumor immune response as peripheral immune organs. The results in the study showed that the pathological type of follicular adenocarcinoma, tumor size ≥2 cm, clinical stage of III~IV, distant metastasis, and high TSH level before ^125^I particle treatment were all risk factors related to the efficacy of ^125^I particle treatment of TC (*P* < 0.05). Therefore, our study suggested that the increase of immunotoxicity related to the increase of radiotherapy dose might be an important factor leading to tumor progression. It is of great significance to limit the radiation dose to minimize the damage of the immune system for improving the survival of patients.

In general, the efficacy of low-dose and high-dose ^125^I particles in the treatment of differentiated thyroid cancer is comparable, among which low-dose ^125^I particles have fewer adverse effects and have less impact on the immunity of the body, which is well tolerated by patients and can be widely used in clinical practice. In addition, the pathological type of follicular adenocarcinoma, tumor size ≥ 2 cm, clinical stage III~IV, distant metastasis, and high TSH level before ^125^I particle treatment are all risk factors that affect the poor effect of ^125^I particles on thyroid cancer treatment, and early monitoring of the above index changes can help evaluate the prognosis. However, due to the limited time of this study, and for patients undergoing radiotherapy and chemotherapy at the same time, the peripheral blood immune indicators may be affected by the radiotherapy. How to control the metrological parameters after ^125^I particle therapy and how to reduce the impact on the body immunity while ensuring the efficacy will be further explored in the following study.

## Figures and Tables

**Table 1:** Comparison of curative effect (cases, %).

Groups	CR	PR	NC	PD	Total effective rate
Low-dose group (*n* = 52)	29 (55.77)	6 (11.54)	6 (11.54)	11 (21.15)	41 (78.85)
High-dose group (*n* = 52)	31 (59.61)	7 (13.46)	5 (9.62)	9 (17.31)	43 (82.69)
*χ* ^2^					0.248
*P*					0.619

**Table 2:** Changes of tumor volume $\left(\bar{x}\pm s\right)$.

Time	Groups	Tumor volume (cm^3^)	Tg (*μ*g/L)
Before treatment	Low-dose group (*n* = 52)	7.15 ± 2.46	57.46 ± 14.29
	High-dose group (*n* = 52)	7.08 ± 1.85	57.82 ± 13.96
*t*		0.164	0.130
*P*		0.870	0.897

After treatment	Low-dose group (*n* = 52)	1.96 ± 0.52^a^	38.59 ± 12.48^a^
	High-dose group (*n* = 52)	1.86 ± 0.26^a^	36.59 ± 15.49^a^
*t*		1.240	0.725
*P*		0.218	0.470

Note: ^a^*P* < 0.05 compared with the same group before treatment; Tg: thyroglobulin.

**Table 3:** Comparison of adverse reactions between the two groups (cases, %).

Time	Groups	Nausea	Radiation gastritis	Radiation parotitis	Neck discomfort
1 week after treatment	Low-dose group (*n* = 52)	6 (11.54%)	2 (3.85%)	3 (5.77%)	8 (15.38%)
	High-dose group (*n* = 52)	15 (28.85%)	9 (17.31%)	10 (19.23%)	17 (32.69%)
*χ* ^2^		4.833	4.981	4.308	4.265
*P*		0.028	0.026	0.038	0.039

1 month after treatment	Low-dose group (*n* = 52)	2 (3.85%)	1 (1.92%)	2 (3.85%)	5 (9.62%)
	High-dose group (*n* = 52)	8 (15.38%)	2 (3.85%)	3 (5.77%)	8 (15.38%)
*χ* ^2^		3.983	0.343	0.210	0.791
*P*		0.046	0.558	0.647	0.374

3 months after treatment	Low-dose group (*n* = 52)	1 (1.92%)	1 (1.92%)	0 (0.00%)	2 (3.85%)
	High-dose group (*n* = 52)	2 (3.85%)	3 (5.77%)	1 (1.92%)	3 (5.77%)
*χ* ^2^		0.343	1.040	1.010	0.210
*P*		0.558	0.308	0.315	0.647

**Table 4:** Comparison of immune function $\left(\bar{x}\pm s\right)$.

Time	Groups	ALC (10^9^/L)	NLR	LMR	PLR
Before treatment	Low-dose group (*n* = 52)	1.85 ± 1.26	2.43 ± 1.85	3.63 ± 2.15	152.63 ± 35.85
	High-dose group (*n* = 52)	1.82 ± 1.38	2.40 ± 1.26	3.81 ± 2.45	148.67 ± 46.33
*t*		0.116	0.096	0.398	0.488
*P*		0.908	0.923	0.691	0.627

After treatment	Low-dose group (*n* = 52)	0.91 ± 0.46^a^	3.59 ± 2.15^a^	2.48 ± 1.26^a^	225.46 ± 102.53^a^
	High-dose group (*n* = 52)	0.81 ± 0.37^a^	6.36 ± 5.12^a^	1.69 ± 1.05^a^	316.48 ± 142.03^a^
*t*		1.222	3.597	3.473	3.747
*P*		0.225	0.001	0.001	<0.001

Note: ^a^*P* < 0.05 compared with the same group before treatment; ALC: absolute lymphocyte count; NLR: neutrophil to lymphocyte ratio; LMR: lymphocyte to monocyte ratio; PLR: platelet to lymphocyte ration.

**Table 5:** Univariate analysis of factors influencing the efficacy of ^125^I particle therapy for differentiated TC.

Related factors	Effective group (*n* = 85)	Ineffective group (*n* = 19)	*χ* ^2^	*P*
Gender				
Male	33 (38.82)	11 (57.89)	2.314	0.128
Female	52 (61.18)	8 (42.11)		
Age				
<45 years old	47 (55.29)	7 (36.84)	2.118	0.146
≥45 years old	38 (44.71)	12 (63.16)		
Pathological type				
Papillary adenocarcinoma	51 (60.00)	5 (26.32)	7.090	0.008
Follicular adenocarcinoma	34 (40.00)	14 (73.68)		
Tumor size				
<2 cm	63 (74.18)	7 (36.84)	9.806	0.002
≥2 cm	22 (25.88)	12 (63.16)		
Number of lesions				
Single	27 (31.76)	9 (47.38)	1.671	0.196
Multiple	58 (68.24)	10 (52.63)		
Invasion				
No invasion of capsule	30 (35.29)	5 (26.32)	0.974	0.808
Membrane invasion	27 (31.76)	8 (42.11)		
Slight invasion outside the thyroid	16 (18.82)	3 (15.79)		
Obvious invasion outside the thyroid	12 (14.12)	3 (15.79)		
Clinical stage				
I~II stage	60 (70.59)	8 (42.11)	5.566	0.018
III~IV stage	25 (29.41)	11 (57.89)		
Distant metastasis				
Yes	30 (35.29)	13 (68.42)	7.027	0.008
No	55 (64.71)	6 (31.58)		
TSH level before ^125^I particle therapy (mIU/L)				
<30	1 (1.18)	2 (10.53)	7.896	0.048
30~59	37 (43.53)	4 (21.05)		
60~89	24 (28.24)	5 (26.32)		
≥90	23 (27.06)	8 (42.11)		

Note: TSH: thyroid-stimulating hormone.

**Table 6:** Risk factors related to the efficacy of ^125^I particle treatment analyzed by multivariate logistic regression analysis.

Factors	*B*	SE	Wald	*P*	OR	95% CI
Follicular adenocarcinoma	0.985	0.263	12.218	0.001	2.718	1.469~4.852
Tumor size ≥ 2 cm	1.236	0.318	5.006	0.001	3.421	2.052~5.526
Clinical stage of III~IV	1.181	0.283	6.775	0.001	3.283	1.562~6.571
Distant metastasis	0.860	0.363	5.136	0.002	2.359	1.182~4.203
TSH level ≥ 90	0.952	0.413	5.750	0.001	2.523	1.409~4.853

Note: TSH: thyroid-stimulating hormone.

## Data Availability

The datasets used and/or analyzed during the current study are available from the corresponding author on reasonable request.
